# Association of Plasma Anion Gap with 28-Day Inhospital Mortality and 1-Year Mortality of Patients with Alcohol Use Disorder at ICU Admission: A Retrospective Cohort Study

**DOI:** 10.1155/2022/5039964

**Published:** 2022-09-07

**Authors:** Yu Pan, Xiaoyang Miao, Ou Jin, Jingye Pan, Yihua Dong

**Affiliations:** ^1^Department of Pharmacy, Wenzhou Hospital of Integrated Traditional Chinese and Western Medicine, Wenzhou, Zhejiang 325000, China; ^2^Department of Intensive Care Unit, The First Affiliated Hospital of Wenzhou Medical University, Wenzhou, Zhejiang 325000, China

## Abstract

**Background:**

Alcohol use disorder (AUD) is common in critically ill patients. Plasma anion gap (AG) was known as a feasible parameter and was associated with outcomes of various diseases. This study is intended to explore whether AG is related to 28-day inhospital mortality and 1-year mortality of critically ill patients with AUD.

**Method:**

We extracted data from the Medical Information Mart for Intensive Care IV (MIMIC-IV) database. The association of plasma AG with 28-day inhospital mortality and 1-year mortality of critically ill AUD patients was assessed using Cox proportional hazard regression models and stratification analyses, allowing AG as a time-varying covariate in the models. To evaluate the accuracy of AG in predicting different endpoints, receiver operator characteristic (ROC) curves were used.

**Result:**

Among the 3993 critically ill patients with AUD, AG was positively associated with 28-day inhospital mortality and 1-year mortality after adjusting confounders (*p* < 0.001 for all). Compared with lower AG (<12 mmol/L), patients in different groups (12 ≤ AG < 14 mmol/L, 14 ≤ AG < 17 mmol/L, 17 ≤ AG < 20 mmol/L, and AG ≥ 20 mmol/L) had different HRs (95% CIs) for 28-day inhospital mortality (1.105, (0.906, 1.347); 1.171, (0.981, 1.398); 1.320, (1.108, 1.573); and 1.487, (1.254, 1.763), respectively) and 1-year mortality (1.037 (0.898, 1.196); 1.091 (0.955, 1.246); 1.201 (1.052, 1.371); and 1.3093 (1.149, 1.492), respectively).

**Conclusion:**

Increased AG is associated with greater 28-day inhospital mortality and 1-year mortality. The effect of AG on all-cause mortality is linear in critically ill AUD patients.

## 1. Introduction

Alcohol use disorder (AUD) is a chronic disease characterized by unhealthy patterns of alcohol use and impaired ability to stop or control it [1]. It has been reported that the prevalence rate of AUD is approximately 5.1% in the worldwide [2]. According to the statistics, the number of people with AUD is about 76.3 million worldwide, with 1.8 million deaths per year [3]. AUD is one of the most prevalent mental disorders in the world. In 2017, a national epidemiological survey of US adults showed that the prevalence of AUD increased by 49.4% from 8.5% to 12.7% between 2001-2002 and 2012-2013 [4]. AUD can cause significant clinical impairment such as liver disease [5], heart and blood vessel disease [6], and brain disease [7]. AUD is common in critically ill patients and approximately 16-31% of them are admitted to the intensive care unit [8]. These patients tend to have a higher risk of serious illness, longer ICU stays, and more complications, leading to greater mortality [9]. It creates such a stressful burden on the home caregivers, the society, and our healthcare system [10].

The anion gap (AG) is a feasible parameter, which is commonly used to evaluate acid-base balance and can be used as a marker of various metabolic acidosis [11]. AG is also an important biomarker commonly used in clinical practice for the diagnosis or prognosis of many diseases [12–15]. Previous studies have shown that there is a positive relationship between high AG and mortality in several diseases, such as coronary artery disease (CAD) [13], acute kidney injury (AKI) [16], sepsis [17], cardiogenic shock (CS) [18], and admission to the intensive care unit (ICU) [19].

As we know, no epidemiological study has investigated the association between AG and all-cause mortality of critically ill AUD patients. Therefore, in this study, we aimed to investigate the relationship between AG and all-cause mortality in these patients.

## 2. Methods

### 2.1. Data Source

The data analyzed in this study were extracted from the MIMIC-IV database [20]. MIMIC-IV, an update to MIMIC-III, is a publicly and freely available database. This database contains clinical information of patients in the intensive care unit at Beth Israel Deaconess Medical Center (BIDMC) from 2008 to 2019. One author, Yihua Dong, has completed the Collaborative Institutional Training Initiative examination (certification number: 22691479) and achieved access to the database for data extraction. Since this was a retrospective cohort study, informed consent was not required.

### 2.2. Population Selection Criteria

We conducted the retrospective cohort study on adult patients (age ≥ 16 years) with the diagnosis of AUD at the first administration. ICD-9 diagnostic codes (291.xx, 303.xx, 305.0x, 357.5, 425.5, 535.30, 535.31, 571.0, 571.1, 571.2, and 571.3) were used to identify AUD patients, except for ICD-9 diagnostic codes 303.03, 303.93, or 305.03 [21].

Patients who stayed in the ICU for less than 24 hours were excluded. If a patient had multiple administration records or ICU stay records, we only took the first ICU administration record. Patients without AG values were also not included.

### 2.3. Data Extraction

Our data were extracted from the database using structured query language (SQL) with Navicat and open codes from the GitHub website of Alistairewj's homepage (https://github.com/MIT-LCP/mimic-iv). Patient identifiers, comorbidities, laboratory parameters, scoring systems, and vital signs recorded within 24 h upon admission to ICU were collected directly or calculated with the data from the database. The severity scoring systems were recorded for each patient including Logistic Organ Dysfunction System (LODS), Sequential Organ Failure Assessment (SOFA) score, Simplified Acute Physiology Score II (SAPSII), Quick Sepsis-Related Organ Failure Assessment (QSOFA) score, and Systemic Inflammatory Response Syndrome (SIRS) score. Our outcomes were set as 28-day inhospital mortality and 1-year mortality.

### 2.4. Statistical Analysis

Continuous variables were presented as median and interquartile range (IQR) and compared using the Kruskal-Wallis test. Categorical variables were expressed as numbers and percentages, and Chi-square test or Fisher's test was used for comparisons between groups.

To evaluate the diagnostic performance of AG, LODS, SOFA, QSOFA, SIRS, SAPSII, and their combination in predicting 28-day inhospital mortality and 1-year mortality of AUD, the receiver operating characteristic (ROC) curve analysis was applied. We obtained the coefficient (*β*) of all variables by using a logistic regression model and calculated a new variable *Y* according to the equation: *Y* = exp (*β*0 + *β*1*X*1 + *β*2*X*2+⋯+*βnXn*)/1 + exp (*β*0 + *β*1*X*1 + *β*2*X*2+⋯+*βnXn*) [22], where *X* represents the variables: AG, LODS, SOFA, QSOFA, SIRS, and SAPSII.

Patients were grouped into quintiles by the AG according to cutoffs derived from clinical experience and literatures [16, 18, 23, 24]. The Kaplan-Meier curves were presented for survival analysis, and log-rank tests were used to compare survival rates between groups. We constructed univariable and multivariable Cox hazard regression models with the time-varying risk effect to estimate the association of AG with 28-day inhospital mortality and 1-year mortality. These results were expressed as HRs with 95% CIs. Potentially significant confounders (*p* < 0.1) in univariable analysis were entered into multivariable Cox regression models. The Hosmer-Lemeshow test was used to evaluate the suitability of the regression model. We conducted a sensitivity analysis to ensure the robustness of our analysis.

The time dependence effects of AG and other variables in the models should be considered because they may change during ICU stay. The effect of each variable was modeled using a Cox proportional hazard model, which produced a constant HR over the entire follow-up period, and then, the proportional hazard assumption of the variable was examined. When the proportional hazard assumption was not fulfilled, time-varying effect emerged. *p* < 0.05 indicated that there was significant deviation from the proportion hazard assumption and the variable violated the proportion hazard assumption. In general, the global *p* value is derived from a relevant global significance test, and *p* < 0.05 indicates that the model is not fit [25].

Finally, we employed subgroup analyses to evaluate the association between the time-varying AG levels and all-cause mortality, including age, gender, scoring systems, comorbidities, and laboratory parameters. The likelihood ratio test was used to estimate the significance of the interaction. All statistical analyses were performed using statistical packages R (version 4.0.5). A two-tailed *p* < 0.05 was considered to be statistically significant.

## 3. Results

### 3.1. Patient Characteristics

**Figure 1:**
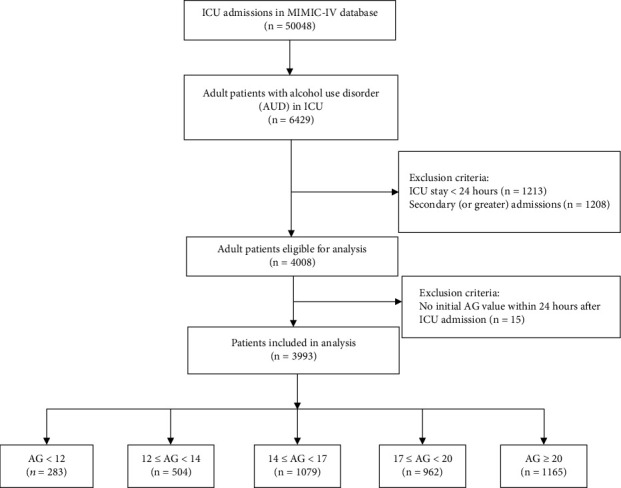
Study chart.

A total of 3993 patients who fulfilled the inclusion and exclusion criteria mention above were enrolled in this study (Figure 1).  Table 1 summarizes the baseline characteristics of all the study patients stratified by their AG. In general, the median age of the entire cohort was 55.00 years (45.00-64.00), and about 73.13% of them were male. The most common comorbidities were hepatic disease (62.61%) and sepsis (57.02%). The overall 28-day inhospital all-cause mortality of the whole cohort was 11.24%. The basic demographic characteristics of the 449 patients who died in the hospital within 28 days (28-day inhospital nonsurvivors) compared to the 28-day inhospital survivors were shown in Table S1. The 28-day inhospital nonsurvivors had a higher AG and score of SAPSII, LODS, and SOFA than the survivors (*p* < 0.001 for all).

According to the AG, 283, 504, 1079, 962, and 1165 patients belonged to the first quintile (<12), second quintile (12 ≤ AG < 14), third quintile (14 ≤ AG < 17), fourth quintile (17 ≤ AG < 20), and fifth quintile (≥20), respectively. Patients with AG ≥ 17 were more likely to have comorbidities of renal disease while patients with AG < 17 were more tend to be complicated with chronic pulmonary disease. Patients with AG ≥ 20 had higher SAPSII, LODS, and SOFA and had more comorbidities such as sepsis, mild liver disease, and severe liver disease. And 28-day inhospital mortality showed a tendency of increasing with the AG quintiles as well as 1-year mortality (*p* < 0.001 for all).

### 3.2. Predictive Values of AG and Some Severity Scoring Systems for 28-Day Inhospital Mortality and 1-Year Mortality

**Figure 2:**
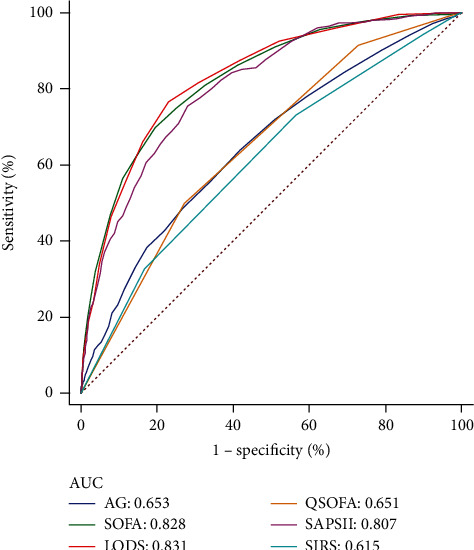
ROC curve for 28-day inhospital mortality.

Some severity scoring systems, such as LODS, SAPSII, SOFA, SIRS, and QSOFA, were a scoring tool that provided a potential prediction of the mortality. The predictive values of AG and some severity scoring systems for 28-day inhospital mortality and 1-year mortality are listed in Table S2. In terms of 28-day inhospital mortality, the predictive values were as follows: SIRS (area under ROC: 0.615, 95% CI: 0.600-0.630), QSOFA (area under ROC: 0.651, 95% CI: 0.636-0.666), AG (area under ROC: 0.653, 95% CI: 0.638-0.668), SAPSII (area under ROC: 0.807, 95% CI: 0.795-0.820), SOFA (area under ROC: 0.828, 95% CI: 0.816-0.839), and LODS (area under ROC: 0.831, 95% CI: 0.819-0.843). Compared with QSOFA or SIRS alone, the area under ROC of each severity score combined with AG was significantly higher in predicting 28-day inhospital mortality and 1-year mortality (*p* < 0.0001 for all). The combination of these variables significantly improved the discriminatory ability (area under ROC: 0.853, 95% CI: 0.841-0.863). The predictive ability of severity scoring systems and AG to predict 28-day inhospital mortality is shown in Figure 2.

### 3.3. Association of AG with 28-Day Inhospital Mortality and 1-Year Mortality

The univariable and multivariable time-varying covariate Cox hazard regression models were built to investigate the association of AG with 28-day inhospital mortality and 1-year mortality. Time-varying adjusted HRs for 28-day inhospital mortality and 1-year mortality were 1.013 (95% CI: 1.010-1.017) and 1.010 (95% CI: 1.007-1.013), respectively (*p* < 0.0001 for all). The continuous variable of AG was converted to categorical variable according to quintiles and cutoffs derived from clinical experience and literatures. We used the first category AG as the reference group to assess whether increased AG was associated with different endpoints in the association analyses. Potential confounders were adjusted in model 2 (Adjust I) and model 3 (Adjust II). We evaluated the proportional hazard assumption for each factor in the multivariable regression models. The results showed that factors such as time in ICU, ventilation, and vasopression violated the proportional hazard assumption (*p* < 0.05 for all), and their time-varying effects were considered. The trend of the effect size in different AG groups was consistent with the *p* for trend of AG with 28-day inhospital mortality and 1-year mortality (*p* < 0.0001). In the adjusted models, higher AG level was linearly associated with higher risk of 28-day inhospital mortality (model 3: time-varying adjusted HR, 95% CI: 1.105, 0.906-1.347; 1.171, 0.981-1.398; 1.320, 1.108-1.573; and 1.487, 1.254-1.763) (Table 2). Similarly, our findings showed a similar trend between AG and 1-year mortality (Table 3).

**Figure 3:**
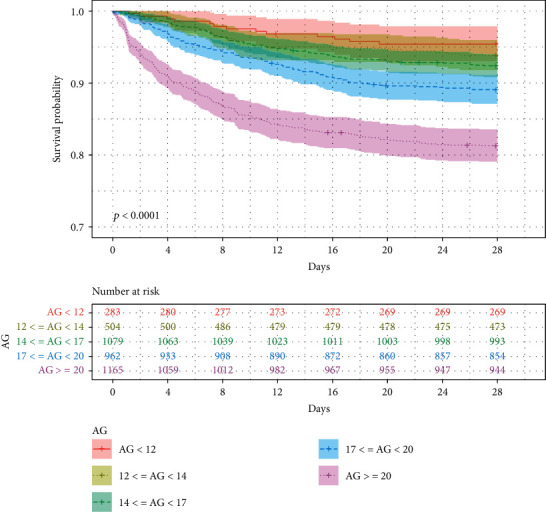
Kaplan-Meier curve of 28-day inhospital mortality for overall survival analysis of different AG categories.

**Figure 4:**
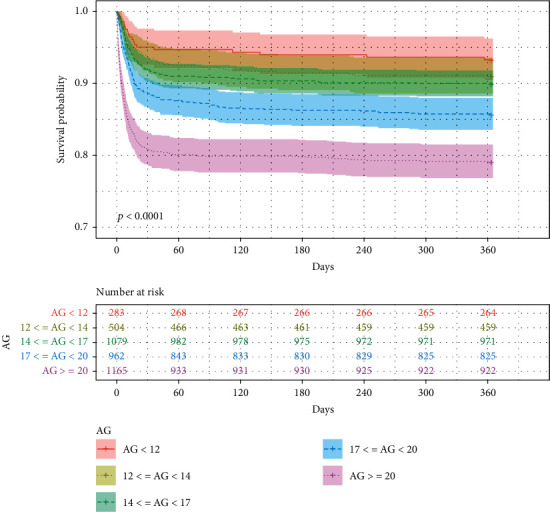
Kaplan-Meier curve of 1-year mortality for overall survival analysis of different AG categories.

The associations between the AG category and different endpoints were shown in the Kaplan-Meier survival curves in Figures 3 and 4. The 28-day inhospital (28-day inhospital survival: 95.41% vs. 93.85% vs. 92.40% vs. 89.09% vs. 81.29%, *p* < 0.0001; Figure 3) and 1-year (1-year survival: 93.29% vs. 91.07% vs. 89.99% vs. 85.76% vs. 79.14%, *p* < 0.0001; Figure 4) survival was significantly lower in higher AG category.

### 3.4. Subgroup Analyses

**Figure 5:**
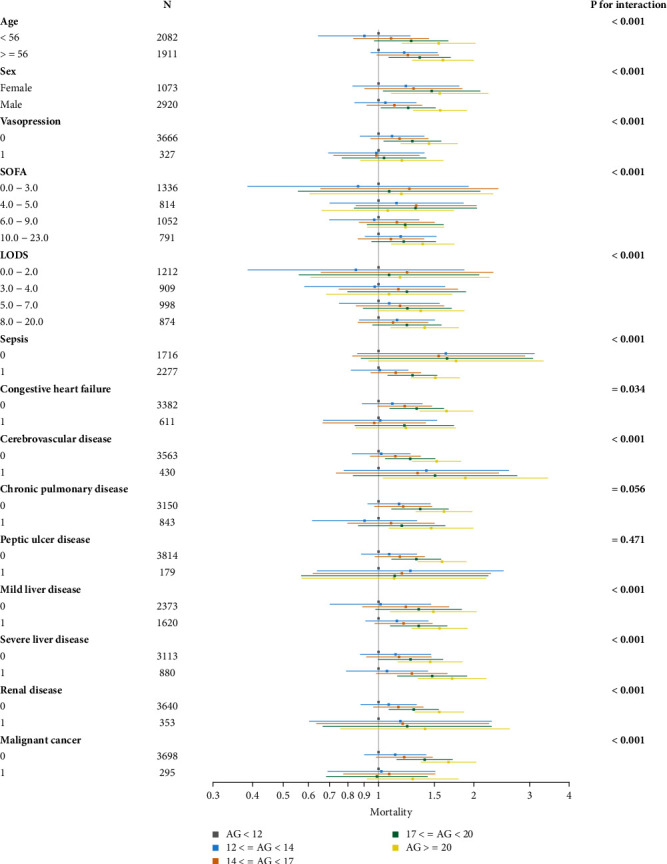
Subgroup analysis of the association between different AG categories and 28-day inhospital mortality.

We performed subgroup analyses to identify the potential factors that influence the impact of AG on 28-day inhospital all-cause mortality (Table S3). Subgroup analysis was based on the following strata: age, gender, SOFA, SAPSII, LODS, and major comorbidities, such as sepsis, congestive heart failure, mild liver disease, and renal disease. In most subgroups, the results in each subgroup population were consistent with the main analysis. Significant changes were detected in age, gender, ventilation, vasopressin, SOFA, SAPSII, LODS, sepsis, congestive heart failure, cerebrovascular disease, mild liver disease, severe liver disease, renal disease, malignant cancer, and metastatic solid tumor. All of these factors had a significant interaction with AG on 28-day inhospital mortality (Figure 5). Severe liver disease was found to have a positive effect, and the AG of patients with severe liver disease had a higher risk of 28-day inhospital mortality (time-varying adjusted HR, 95% CI: 1.021, 1.016-1.025, *p* < 0.0001). Subgroup analysis showed that higher AG levels were positively associated with higher risks of 28-day inhospital mortality in AUD patients with (time-varying adjusted HR, 95% CI: 1.063, 0.791-1.429; 1.274, 0.985-1.647; 1.477, 1.148-1.900; and 1.708, 1.335-2.185) and without severe liver disease (time-varying adjusted HR, 95% CI: 1.132, 0.877-1.462; 1.160, 0.917-1.467; 1.262, 0.998-1.596; and 1.455, 1.151-1.838) (*p* < 0.0001 for all). A similar linear trend was observed in AUD patients with mild liver disease or renal disease. The results of the subgroup analysis for 1-year endpoint are shown in Table S4. There are similar trends of effect sizes in these variables between AG and 1-year mortality.

## 4. Discussion

3993 patients were evaluated to investigate the relationship between AG and 28-day inhospital mortality and 1-year mortality in patients with AUD in this study. To the best of our knowledge, this was the first study to explore the relationship of the AG with all-cause mortality in critically ill patients with AUD.

We found that the AG of critically ill AUD patients who died within 28 days or 1 year of ICU admission was significantly higher than those who survived. After considering the time-varying effect and adjusting multiple potential covariates, higher AG measured in 24 hours after admission to ICU was significantly associated with greater 28-day inhospital mortality and 1-year mortality of patients with AUD compared with the first quintile (AG < 12). We also found that the effect of AG on all-cause mortality is linear in critically ill AUD patients.

AG is a traditional factor for evaluating acid-base states of patients; its abnormal levels often indicates an acid-base imbalance, which has an obvious impact on mortality in critically ill patients [26]. Because it is easily obtained by calculating the plasma concentration of anions, especially in areas with poor medical resources, AG may have a good predictive value for mortality of intensive care patients [27].

Many studies have focused on the relationship between AG and clinical outcomes of various diseases. Zhang et al. [18] studied the association between the AG and mortality in critically ill patients with cardiogenic shock (CS) and suggested that higher AG was related to increased risk of 30-day, 90-day, and 1-year all-cause mortality, HRs (95% CIs) were as follows: 1.62 (1.14–2.30), 1.35 (1.04-1.84), and 1.38 (1.03-1.84), respectively. Gao et al. [24] found that the mortality of aortic aneurysm (AA) in ICU after open surgery increased with increased AG level (OR 1.286, 95% CI: 1.053-1.651) and the growing tendency of mortality was sharper when the AG level was higher than 12 mEq/L. Additionally, Cheng et al. [16] proposed higher AG as a significant predictor of 30-day, 90-day, and 365-day all-cause mortality compared with lower AG (HR, 95% CI: 1.54, 1.33–1.75), 1.55 (1.38-1.73), and 1.46 (1.31-1.60) in critically ill patients with acute kidney injury (AKI). It was found that higher AG levels were associated with increased all-cause mortality. It is widely used to evaluate the acid-base status and is one of the most commonly used biomarkers in clinical practice that provides important clues for the diagnosis and prognosis of various diseases [19, 28]. In our study, we demonstrate that AG was positively associated all-cause mortality in critically ill AUD patients.

AUD is one of the main causes of preventable disease and liver disease-associated mortality, leading to a range of physical injuries. Previous literatures have explored the early risk factors of adverse events in AUD patients. Lin and Liao [29] studied relationship of red blood cell distribution width (RDW) with 28-day mortality in critically ill patients with AUD and suggested that RDW > 15.45% was associated with increased 28-day mortality (HR 1.964, 95% CI: 1.429-2.698). Fuster et al. [30] assessed the association between three baseline markers of inflammation (anemia, fibrinogen, and ferritin levels) and all-cause mortality of patients with alcohol dependence. The results showed that in the multivariable analysis, the presence of anemia at admission was associated with all-cause mortality in patients with alcohol dependence (HR 1.67, 95% CI: 1.11-2.52), while fibrinogen (fibrinogen > 4.5 mg/dL: HR 1.27, 95% CI: 0.82-1.97) and ferritin (ferritin > 200 ng/mL: HR 1.32, 95% CI: 0.87-2.00) levels were not related to the mid-term mortality in the unadjusted analysis. Moreover, the association between lactate and 30-day mortality in critically ill patients with AUD was also investigated [31]. When compared to the reference group (lactate < 1.3 mmol/L), the second (1.3 mmol/L ≤ lactate < 2 mmol/L), and third (lactate ≥ 2 mmol/L) levels were statistically significant risk factors for 30-day all-cause mortality after adjusted variables (the second group: HR 1.6, 95% CI 1.0-2.6; the third group: HR 2.7, 95% CI: 1.7-4.4).

There are usually two causes for AG elevation: increased production of acid anions and/or decreased anion excretion [32], which is mainly caused by the increase of serum lactate and ketone anions [28] and is often found among some diseases, such as lactic acidosis, ketosis, sepsis, renal failure, or poisoning [26], and these conditions are common in AUD patients. Excessive alcohol consumption leads to increased levels of acetaldehyde and harmful prooxidants by increasing the production of enzymes needed for metabolism. These factors can affect the metabolic function of the liver and hinder the metabolism of lactate, which may lead to the increase of AG level in AUD patients. In addition, when AUD patients are under poor nutritional status and their oxidation reduction state changes, alcoholic ketoacidosis (AKA) can be caused. And AKA may cause an anion gap metabolic acidosis (AGMA) and an elevated osmol gap [33].

Critically ill patients often suffer from hypoxia and anoxia, which can lead to a rapid accumulation of pyruvate and eventually almost conversion to lactic acid [34]. The possible reason for metabolic acidosis caused by elevated blood lactic acid level may be that alcohol over consumption in critically ill patients with AUD can increase NADH/NAD ratio and promote pyruvate metabolism into lactic acid. In addition, critically ill patients with AUD often suffer from disorders of alcohol metabolism, abnormal lipid metabolism, inflammation, oxidative stress, and ion channel opening. These mechanisms can cause multiple organs damage [35], especially liver disease and renal dysfunction, which will inevitably lead to blocked excretion of lactic acid and other substances, and then metabolic acidosis. Consequently, high AG is common in critically ill AUD patients. These strongly suggest that high AG may be related to the prognosis in critically ill AUD patients and that increased AG level at ICU admission indicate potential acidosis, which ultimately leads to worse prognosis and higher mortality. Therefore, our results support the above hypothesis.

A meta-analysis showed that AG alone could not be used as a predictor of mortality in critically ill patients, and most of the studies included did not support serum AG as a predictor of 31-day mortality, ICU mortality, or inhospital mortality [27]. Similarly, the results of our study showed that AG had moderate predictive power in predicting all-cause mortality. We found that the predictive value of AG was worse than that of SOFA, SAPSII, and LODS in predicting 28-day inhospital mortality and 1-year mortality of critically ill AUD patients, but significantly higher than that of QSOFA and SIRS. Compared with QSOFA or SIRS alone, the AUC of each score system combined with AG was significantly higher in predicting all-cause mortality. The combination of these variables significantly improved the discriminatory ability.

We found that the relationship between AG level and 28-day inhospital mortality and 1-year mortality for AUD patients with severe liver disease was significantly stronger than that for AUD patients without severe liver disease. And higher AG levels were positively associated with greater risks of 28-day inhospital mortality in AUD patients with and without severe liver disease. A similar trend was observed in AUD patients with mild liver disease or renal disease. The possible cause may be that chronic alcohol consumption is a main cause of chronic liver diseases, resulting in alcoholic hepatitis, fibrosis/cirrhosis, and hepatocellular carcinoma. Ceni et al. [36] highlighted the role of alcohol abuse in liver disease by examining ethanol metabolism. Alcohol consumption is a risk factor for kidney injury, although the underlying mechanism is still largely unknown [37]. Chronic alcoholism can cause kidney damage, which leads to increased mortality in patients with alcoholic hepatitis [38]. As mentioned above, higher AG levels are associated with increased all-cause mortality in critically ill patients with acute kidney injury [16].

There are several limitations in our study. First of all, this study was a retrospective study with samples from a public database, and selection bias may influence the results. Secondly, the study sample contained heterogeneous subphenotypes, and the predictive function of AG was different from subgroups. Although metabolic dysfunction is the main reason for the AG increased in critically ill AUD patients, the complexity and dynamic interaction of different factors (such as gender, the existence of genetic variation, different comorbidities, and different levels of alcohol consumption) determine the different physical condition and prognosis of disease subphenotypes and eventually may produce different reaction to AG. Thirdly, we measured plasma AG only upon admission to the ICU and no dynamic data from the following day. We considered the time-varying effect of AG and analyzed it with Cox regression model, which may reduce its influence on the summary results. Finally, we had no specific data on the causes of death, so we cannot illustrate other causes of death. Therefore, the relationship between AG and the mortality of critically ill patients with AUD needs to be further clarified in a more rigorous study.

## 5. Conclusions

We confirmed that AG was positively associated with all-cause mortality of critically ill patients with AUD in the present study. Increased AG in critically ill patients with AUD is associated with greater 28-day inhospital mortality and 1-year mortality. The effect of AG on all-cause mortality is linear in critically ill AUD patients. AG, as an effective auxiliary tool, can improve the ability to predict the clinical outcome of critically ill AUD patients to a certain extent.

## Figures and Tables

**Table 1:** Baseline characteristics between AG groups.

Variable	AG (mmol/L)					
	AG < 12	12 ≤ AG < 14	14 ≤ AG < 17	17 ≤ AG < 20	AG ≥ 20	*p* value
	(*n* = 283)	(*n* = 504)	(*n* = 1079)	(*n* = 962)	(*n* = 1165)	
Age (years)	58.00 (48.00-66.00)	56.00 (47.00-65.00)	55.00 (45.00-64.00)	54.00 (44.00-64.00)	54.00 (45.00-62.00)	<0.001
Sex (male), *n* (%)	209 (73.85%)	388 (76.98%)	788 (73.03%)	683 (71.00%)	852 (73.13%)	0.191
Time in ICU (days)	2.29 (1.49-4.72)	2.53 (1.57-5.16)	2,63 (1.58-5.34)	2.73 (1.71-6.01)	2.79 (1.70-6.04)	0.002
Time in hospital (days)	7.96 (5.08-12.83)	7.73 (4.59-14.26)	8.29 (4.71-14.96)	8.23 (4.58-15.54)	8.42 (4.17-15.58)	0.803
28-day inhospital mortality, *n* (%)	13 (4.59%)	31 (6.15%)	82 (7.60%)	105 (10.91%)	218 (18.71%)	<0.001
1-year mortality, *n* (%)	19 (6.71%)	45 (8.93%)	108 (10.01%)	137 (14.24%)	243 (20.86%)	<0.001
Vital signs						
Anion gap	10.00 (10.00-11.00)	13.00 (12.00-13.00)	15.00 (14.00-16.00)	18.00 (17.00-19.00)	23.00 (21.00-27.00)	<0.001
Scoring systems						
SOFA	5.00 (3.00-7.00)	4.00 (2.00-7.00)	4.00 (2.00-7.00)	5.00 (3.00-8.00)	7.00 (3.00-11.00)	<0.001
LODS	4.00 (2.00-6.00)	3.00 (2.00-6.00)	4.00 (2.00-6.00)	4.00 (2.00-7.00)	5.00 (3.00-9.00)	<0.001
SAPSII	29.00 (22.00-36.00)	28.00 (21.00-37.00)	29.00 (21.00-37.00)	31.00 (22.00-41.00)	35.00 (25.00-48.00)	<0.001
QSOFA, *n* (%)						0.001
0	6 (2.12%)	13 (2.58%)	27 (2.50%)	27 (2.81%)	23 (1.97%)	
1	67 (23.67%)	121 (24.01%)	251 (23.26%)	196 (20.37%)	268 (23.00%)	
2	144 (50.88%)	241 (47.82%)	489 (45.32%)	468 (48.65%)	468 (40.17%)	
3	66 (23.32%)	129 (25.60%)	312 (28.92%)	271 (28.17%)	406 (34.85%)	
SIRS, *n* (%)						<0.001
0	7 (2.47%)	11 (2.18%)	14 (1.30%)	6 (0.62%)	6 (0.52%)	
1	42 (14.84%)	53 (10.52%)	100 (9.27%)	79 (8.21%)	63 (5.41%)	
2	90 (31.80%)	179 (35.52%)	351 (32.53%)	335 (34.82%)	332 (28.50%)	
3	103 (36.40%)	192 (38.10%)	431 (39.94%)	359 (37.32%)	332 (28.50%)	
4	41 (14.49%)	69 (13.69%)	183 (16.96%)	183 (19.02%)	262 (22.49%)	
Ethnicity, *n* (%)						<0.001
White	195 (68.90%)	318 (63.10%)	669 (62.00%)	612 (63.62%)	700 (60.09%)	
Black	12 (4.24%)	25 (4.96%)	94 (8.71%)	87 (9.04%)	141 (12.10%)	
Hispanic	7 (2.47%)	26 (5.16%)	45 (4.17%)	39 (4.05%)	50 (4.29%)	
Others	69 (24.38%)	135 (26.79%)	271 (25.12%)	224 (23.28%)	274 (23.52%)	
First care unit, *n* (%)						<0.001
MICU	87 (30.74%)	146 (28.97%)	322 (29.84%)	318 (33.06%)	481 (41.29%)	
CCU	62 (21.91%)	87 (17.26%)	218 (20.20%)	181 (18.81%)	172 (14.76%)	
SICU	33 (11.66%)	82 (16.27%)	173 (16.03%)	150 (15.59%)	166 (14.25%)	
MICU/SICU	14 (4.95%)	36 (7.14%)	68 (6.30%)	72 (7.48%)	87 (7.47%)	
CVICU	31 (10.95%)	70 (13.89%)	171 (15.85%)	158 (16.42%)	188 (16.14%)	
TSICU	54 (19.08%)	57 (11.31%)	78 (7.23%)	28 (2.91%)	25 (2.15%)	
Others	2 (0.71%)	26 (5.16%)	49 (4.54%)	55 (5.72%)	46 (3.95%)	
Ventilation, *n* (%)	237 (83.75%)	421 (83.53%)	863 (79.98%)	732 (76.09%)	912 (78.28%)	0.003
Vasopression, *n* (%)	8 (2.83%)	19 (3.77)	57 (5.28%)	76 (7.90%)	167 (14.33%)	<0.001
Major comorbidities, *n* (%)						
Sepsis	156 (55.12%)	292 (57.94%)	575 (53.29%)	523 (54.37%)	731 (62.75%)	<0.001
Myocardial infarct	30 (10.60%)	43 (8.53%)	95 (8.80%)	84 (8.73%)	107 (9.18%)	0.877
Congestive heart failure	33 (11.66%)	73 (14.48%)	184 (17.05%)	148 (15.38%)	173 (14.85%)	0.207
Peripheral vascular disease	28 (9.89%)	33 (6.55%)	77 (7.14%)	66 (6.86%)	56 (4.81%)	0.019
Cerebrovascular disease	19 (6.71%)	55 (10.91%)	135 (12.51%)	128 (13.31%)	93 (7.98%)	<0.001
Peptic ulcer disease	16 (5.65%)	24 (4.76%)	48 (4.45%)	34 (3.53%)	57 (4.89%)	0.481
Mild liver disease	122 (43.11%)	202 (40.08%)	362 (33.55%)	353 (36.69%)	581 (49.87%)	<0.001
Severe liver disease	76 (26.86%)	114 (22.62%)	194 (17.98%)	181 (18.81%)	315 (27.04%)	<0.001
Chronic pulmonary disease	64 (22.61%)	128 (25.40%)	246 (22.80%)	200 (20.79%)	205 (17.60%)	0.002
Renal disease	11 (3.89%)	27 (5.36%)	65 (6.02%)	100 (10.40%)	150 (12.88%)	<0.001
Metastatic solid tumor	9 (3.18%)	12 (2.38%)	36 (3.34%)	21 (2.18%)	26 (2.23%)	0.398
Malignant cancer	29 (10.25%)	41 (8.13%)	91 (8.43%)	60 (6.24%)	74 (6.35%)	0.056
Aids	2 (0.71%)	3 (0.60%)	10 (0.93%)	9 (0.94%)	9 (0.77%)	0.952

Abbreviations: AG: anion gap; CCU: cardiac care unit; CVICU: cardiovascular intensive care unit; ICU: intensive care unit; LODS: logistic organ dysfunction system; MICU: medical intensive care unit; QSOFA: quick sepsis-related organ failure assessment; SAPSII: simplified acute physiology score II; SICU: surgical intensive care unit; SIRS: systemic inflammatory response syndrome; SOFA: sequential organ failure assessment; TSICU: trauma and surgical intensive care unit.

**Table 2:** Time-varying HRs and 95% CI for AG in association with 28-day inhospital mortality from Cox models.

	Nonadjusted		Adjusted I		Adjusted II	
	HR (95% CI)	*p*	HR (95% CI)	*p*	HR (95% CI)	*p*
AG	1.022 (1.018, 1.026)	<0.0001	1.023 (1.020, 1.027)	<0.0001	1.013 (1.010, 1.017)	<0.0001
AG^Quintile^						
AG < 12	1		1		1	
12 ≤ AG < 14	1.092 (0.901, 1.324)	0.3700	1.100 (0.906, 1.335)	0.3348	1.105 (0.906, 1.347)	0.3265
14 ≤ AG < 17	1.167 (0.981, 1.387)	0.0818	1.189 (0.999, 1.415)	0.0509	1.171 (0.981, 1.398)	0.0805
17 ≤ AG < 20	1.305 (1.099, 1.550)	0.0024	1.321 (1.112, 1.568)	0.00153	1.320 (1.108, 1.573)	0.00191
AG ≥ 20	1.563 (1.320, 1.851)	<0.0001	1.596 (1.347, 1.891)	<0.0001	1.487 (1.254, 1.763)	<0.0001
AG *p* for trend	1.134 (1.104, 1.165)	<0.0001	1.141 (1.111, 1.172)	<0.0001	1.102 (1.073, 1.131)	<0.0001

Abbreviations: AG: anion gap; CI: confidence interval; HR: hazard ratio. Adjusted I for age, gender, and ethnicity. Adjusted II for age, ethnicity, time in hospital, ventilation, vasopression, sepsis, cerebrovascular disease, mild liver disease, peptic ulcer disease, severe liver disease, and metastatic solid tumor. Reference group: AG < 12 mmol/L.

**Table 3:** Time-varying HRs and 95% CI for AG in association with 1-year mortality from Cox models.

	Nonadjusted		Adjusted I		Adjusted II	
	HR (95% CI)	*p*	HR (95% CI)	*p*	HR (95% CI)	*p*
AG	1.016 (1.013, 1.019)	<0.0001	1.018 (1.015, 1.021)	<0.0001	1.010 (1.007, 1.013)	<0.0001
AG^Quintile^						
AG < 12	1		1		1	
12 ≤ AG < 14	1.073 (0.933, 1.234)	0.3230	1.083 (0.941, 1.247)	0.2657	1.037 (0.898, 1.196)	0.62276
14 ≤ AG < 17	1.103 (0.969, 1.256)	0.1370	1.129 (0.992, 1.286)	0.06705	1.091 (0.955, 1.246)	0.20161
17 ≤ AG < 20	1.221 (1.074, 1.388)	0.0022	1.243 (1.093, 1.413)	0.000915	1.201 (1.052, 1.371)	0.006585
AG ≥ 20	1.378 (1.210, 1.569)	<0.0001	1.409 (1.237, 1.605)	<0.0001	1.309 (1.149, 1.492)	<0.0001
AG *p* for trend	1.090 (1.067, 1.114)	<0.0001	1.097 (1.073, 1.120)	<0.0001	1.067 (1.045, 1.090)	<0.0001

Abbreviations: AG: anion gap; CI: confidence interval; HR: hazard ratio. Adjusted I for age, gender, and ethnicity. Adjusted II for age, ethnicity, gender, time in hospital, ventilation, vasopression, sepsis, cerebrovascular disease, peptic ulcer disease, mild liver disease, renal disease, severe liver disease, and metastatic solid tumor. Reference group: AG < 12 mmol/L.

## Data Availability

The clinical data used to support the findings of this study were supplied by Medical Information Mart for Intensive Care IV (MIMIC-IV). All data and material are available at https://mimic.physionet.org/.

## Supplementary Materials

Supplementary Materials Table S1: baseline characteristics of the study population. Table S2: diagnostic performance of AG and scoring systems in predicting 28-day inhospital mortality and 1-year mortality. Table S3: subgroup analysis of the association between time-varying AG and 28-day inhospital mortality. Table S4: subgroup analysis of the association between time-varying AG and 1-year mortality.
