# Supplementary material for: Association of Plasma Anion Gap with 28-Day Inhospital Mortality and 1-Year Mortality of Patients with Alcohol Use Disorder at ICU Admission: A Retrospective Cohort Study
Source: Dis Markers. 2022 Sep 7;2022:5039964. doi: 10.1155/2022/5039964 (PMC9473916; doi:10.1155/2022/5039964)
Supplement: Supplementary Materials — Table S1: baseline characteristics of the study population. Table S2: diagnostic performance of AG and scoring systems in predicting 28-day inhospital mortality and 1-year mortality. Table S3: subgroup analysis of the association between time-varying AG and 28-day inhospital mortality. Table S4: subgroup analysis of the association between time-varying AG and 1-year mortality. [file 5039964.f1.docx]

**Table S1.** Baseline characteristics of the study population.

| Variable | all patients | 28-day in-hospital survivors | 28-day in-hospital non-survivors | P value |
| --- | --- | --- | --- | --- |
|  | (n=3993) | (n=3544) | (n=449) |  |
| Age (years) | 55.00 (45.00-64.00) | 54.00 (45.00-63.00) | 57.00 (50.00-66.00) | <0.001 |
| Sex (Male), n (%) | 2920 (73.13%) | 2601 (73.39%) | 319 (71.05%) | 0.291 |
| Time in ICU (days) | 2.66 (1.66-5.60) | 2.51 (1.60-5.13) | 4.47 (2.18-8.85) | <0.001 |
| Time in hospital (days) | 8.17 (4.58-14.92) | 8.25 (4.71-15.04) | 7.75 (3.50-13.71) | <0.001 |
| Anion gap | 17.00 (14.00-20.00) | 17.00 (14.00-20.00) | 19.00 (16.00-24.00) | <0.001 |
| Scoring systems |  |  |  |  |
| SOFA | 5.00 (3.00-9.00) | 5.00 (2.00-8.00) | 11.00 (7.50-15.00) | <0.001 |
| LODS | 4.00 (2.00-7.00) | 4.00 (2.00-6.00) | 9.00 (7.00-11.00) | <0.001 |
| SAPSII | 31.00 (22.00-41.00) | 29.00 (21.00-38.00) | 46.00 (37.00-58.00) | <0.001 |
| QSOFA, n (%) |  |  |  | <0.001 |
| 0 | 96 (2.40%) | 93 (2.62%) | 3 (0.67%) |  |
| 1 | 903 (22.61%) | 868 (24.49%) | 35 (7.80%) |  |
| 2 | 1810 (45.33%) | 1623 (45.80%) | 187 (41.65%) |  |
| 3 | 1184 (29.65%) | 960 (27.09%) | 224 (49.89%) |  |
| SIRS, n (%) |  |  |  | <0.001 |
| 0 | 44 (1.10%) | 42 (1.19%) | 2 (0.45%) |  |
| 1 | 337 (8.44%) | 313 (8.83%) | 24 (5.35%) |  |
| 2 | 1287 (32.23%) | 1192 (33.63%) | 95 (21.16%) |  |
| 3 | 1587 (39.74%) | 1405 (39.64%) | 182 (40.53%) |  |
| 4 | 738 (18.48%) | 592 (16.70%) | 146 (32.53%) |  |
| Ethnicity, n (%) |  |  |  | <0.001 |
| White | 2494 (62.46%) | 2255 (63.63%) | 239 (53.23%) |  |
| Black | 359 (8.99%) | 331 (9.34%) | 28 (6.24%) |  |
| Hispanic | 167 (4.18%) | 156 (4.40%) | 11 (2.45%) |  |
| Others | 973 (24.37%) | 802 (22.63%) | 171 (38.08%) |  |
| First care unit, n (%) |  |  |  | <0.001 |
| MICU | 1354 (33.91%) | 1144 (32.28%) | 210 (46.77%) |  |
| CCU | 277 (6.94%) | 241 (6.80%) | 36 (8.02%) |  |
| SICU | 604 (15.13%) | 539 (15.21%) | 65 (14.48%) |  |
| MICU/SICU | 618 (15.48%) | 569 (16.06%) | 49 (10.91%) |  |
| CVICU | 242 (6.06%) | 232 (6.55%) | 10 (2.23%) |  |
| TSICU | 720 (18.03%) | 660 (18.62%) | 60 (13.36%) |  |
| Others | 178 (4.46%) | 159 (4.49%) | 19 (4.23%) |  |
| Ventilation, n (%) | 3165 (79.26%) | 2737 (77.23%) | 428 (95.32%) | <0.001 |
| Vasopression, n (%) | 327 (8.19%) | 136 (3.84%) | 191 (42.54%) | <0.001 |
| Major comorbidities, n (%) |  |  |  |  |
| Sepsis | 2277 (57.02%) | 1890 (53.33%) | 387 (86.19%) | <0.001 |
| Myocardial infarct | 359 (8.99%) | 317 (8.94%) | 42 (9.35%) | 0.775 |
| Congestive heart failure | 611 (15.30%) | 533 (15.04%) | 78 (17.37%) | 0.196 |
| Peripheral vascular disease | 260 (6.51%) | 232 (6.55%) | 28 (6.24%) | 0.802 |
| Cerebrovascular disease | 430 (10.77%) | 344 (9.71%) | 86 (19.15%) | <0.001 |
| Peptic ulcer disease | 179 (4.48%) | 162 (4.57%) | 17 (3.79%) | 0.449 |
| Mild liver disease | 1620 (40.57%) | 1308 (36.91%) | 312 (69.49%) | <0.001 |
| Severe liver disease | 880 (22.04%) | 660 (18.62%) | 220 (49.00%) | <0.001 |
| Chronic pulmonary disease | 843 (21.11%) | 747 (21.08%) | 96 (21.38%) | 0.882 |
| Renal disease | 353 (8.84%) | 284 (8.01%) | 69 (15.37%) | <0.001 |
| Metastatic solid tumor | 104 (2.60%) | 82 (2.31%) | 22 (4.90%) | 0.001 |
| Malignant cancer | 295 (7.39%) | 240 (6.77%) | 55 (12.25%) | <0.001 |
| Aids | 33 (0.83%) | 30 (0.85%) | 3 (0.67%) | 0.483 |

Abbreviations: CCU, cardiac care unit; CVICU, cardiovascular intensive care unit; ICU, intensive care unit; LODS, logistic organ dysfunction system; MICU, medical intensive care unit; QSOFA, quick sepsis related organ failure assessment; SAPSII, simplified acute physiology score II; SICU, surgical intensive care unit; SIRS, systemic inflammatory response syndrome; SOFA, sequential organ failure assessment; TSICU, trauma and surgical intensive care unit.

**Table S2.** Diagnostic performance of AG and scoring systems in predicting 28-day in-hospital mortality and 1-year mortality.

|  | 28-day in-hospital mortality | | | |  | 1 year mortality | | | |
| --- | --- | --- | --- | --- | --- | --- | --- | --- | --- |
|  | AUROC (95% CI) | p | Specificity | Sensitivity |  | AUROC (95% CI) | p | Specificity | Sensitivity |
| AG | 0.653 (0.638 - 0.668) |  | 0.583 | 0.639 |  | 0.627 (0.612 - 0.642) |  | 0.661 | 0.525 |
| LODS | 0.831 (0.819 - 0.843) | P = 0.0540 | 0.771 | 0.766 |  | 0.801 (0.788 - 0.813) | P = 0.2183 | 0.776 | 0.694 |
| LODS*AG | 0.835 (0.823 - 0.846) |  | 0.754 | 0.784 |  | 0.803 (0.790 - 0.815) |  | 0.757 | 0.714 |
| SAPSII | 0.807 (0.795 - 0.820) | P = 0.6468 | 0.720 | 0.755 |  | 0.794 (0.781 - 0.806) | P = 0.8504 | 0.729 | 0.719 |
| SAPSII*AG | 0.808 (0.796 - 0.820) |  | 0.707 | 0.760 |  | 0.794 (0.781 - 0.806) |  | 0.650 | 0.795 |
| SOFA | 0.828 (0.816 - 0.839) | P = 0.3036 | 0.806 | 0.697 |  | 0.803 (0.791 - 0.816) | P = 0.5747 | 0.757 | 0.707 |
| SOFA*AG | 0.829 (0.817 - 0.840) |  | 0.768 | 0.744 |  | 0.804 (0.791 - 0.816) |  | 0.779 | 0.692 |
| QSOFA | 0.651 (0.636 - 0.666) | P < 0.0001 | 0.729 | 0.499 |  | 0.636 (0.621 - 0.651) | P < 0.0001 | 0.730 | 0.464 |
| QSOFA*AG | 0.712 (0.698 - 0.726) |  | 0.615 | 0.704 |  | 0.683 (0.668 - 0.697) |  | 0.574 | 0.688 |
| SIRS | 0.615 (0.600 - 0.630) | P < 0.0001 | 0.437 | 0.731 |  | 0.599 (0.583 - 0.614) | P < 0.0001 | 0.439 | 0.714 |
| SIRS*AG | 0.674 (0.659 - 0.688) |  | 0.578 | 0.695 |  | 0.644 (0.629 - 0.659) |  | 0.591 | 0.640 |
| Y | 0.853 (0.841 - 0.863) |  | 0.792 | 0.755 |  | 0.828 (0.816 - 0.840) |  | 0.803 | 0.694 |

Abbreviations: AG, anion gap; AUROC: area under receiver operating curve; LODS, logistic organ dysfunction system; QSOFA, quick sepsis related organ failure assessment; SAPSII, simplified acute physiology score II; SIRS, systemic inflammatory response syndrome; SOFA, sequential organ failure assessment.

Y refers to the combination of AG, LODS, SAPSII, SOFA, QSOFA, and SIRS in predicting all-cause mortality. The regression models are respectively:

28-day in-hospital mortality: Y=exp(-5.249+0.021*AG+0.152*LODS+0.103*SIRS+0.024*SAPSII-0.183*QSOFA+0.123*SOFA)/{1+[exp(-5.249+0.021*AG+0.152*LODS+0.103*SIRS+0.024*SAPSII-0.183*QSOFA+0.123*SOFA)]};

1-year mortality: Y=exp(-4.542+0.010*AG+0.118*LODS+0.076*SIRS+0.029*SAPSII-0.201*QSOFA+0.121*SOFA)/{1+[exp(-4.542+0.010*AG+0.118*LODS+0.076*SIRS+0.029*SAPSII-0.201*QSOFA+0.121*SOFA)]}.

**Table S3. S**ubgroup analysis of the association between time-varying AG and 28-day in-hospital mortality.

|  | N | AG<12 | 12≤AG<14 | 14≤AG<17 | 17≤AG<20 | AG≥20 | p for interaction |
| --- | --- | --- | --- | --- | --- | --- | --- |
|  |  | HR (95%CI) | HR (95%CI) | HR (95%CI) | HR (95%CI) | HR (95%CI) |  |
| Age |  |  |  |  |  |  | <0.001 |
| 18-55 | 2082 | 1.000 (Referent) | 0.902 (0.646, 1.259) | 1.095 (0.833, 1.439) | 1.268 (0.970, 1.658) | 1.549 (1.188, 2.019) |  |
| 56-94 | 1911 | 1.000 (Referent) | 1.205 (0.946, 1.533) | 1.237 (0.988, 1.549) | 1.348 (1.078, 1.686) | 1.597 (1.281, 1.991) |  |
| Sex |  |  |  |  |  |  | <0.001 |
| Female | 1073 | 1.000 (Referent) | 1.220 (0.829, 1.795) | 1.289 (0.905, 1.835) | 1.471 (1.034, 2.092) | 1.560 (1.098, 2.217) |  |
| Male | 2920 | 1.000 (Referent) | 1.051 (0.841, 1.313) | 1.122 (0.919, 1.370) | 1.240 (1.018, 1.511) | 1.565 (1.290, 1.898) |  |
| Ethnicity, n (%) |  |  |  |  |  |  | <0.001 |
| White | 2494 | 1.000 (Referent) | 0.984 (0.740, 1.310) | 1.161 (0.914, 1.474) | 1.391 (1.101, 1.756) | 1.634 (1.295, 2.060) |  |
| Black | 359 | 1.000 (Referent) | 395.7 (0, Inf.) | 293.1 (0, Inf.) | 249.7 (0, Inf.) | 281.8 (0, Inf.) |  |
| Hispanic | 167 | 1.000 (Referent) | 438.7 (0, Inf.) | 249.5 (0, Inf.) | 418.5 (0, Inf.) | 202.0 (0, Inf.) |  |
| Others | 973 | 1.000 (Referent) | 1.080 (0.818, 1.425) | 1.151 (0.892, 1.485) | 1.117 (0.860, 1.451) | 1.527 (1.190, 1.959) |  |
| Ventilation |  |  |  |  |  |  | <0.001 |
| 0 | 828 | 1.000 (Referent) | 0.001 (0, Inf.) | 0.740 (0.4807, 1.138) | 0.788 (0.519, 1.194) | 0.756 (0.502, 1.139) |  |
| 1 | 3165 | 1.000 (Referent) | 1.181 (0.957, 1.458) | 1.254 (1.032, 1.523) | 1.425 (1.175, 1.727) | 1.726 (1.426, 2.089) |  |
| Vasopression |  |  |  |  |  |  | <0.001 |
| 0 | 3666 | 1.000 (Referent) | 1.103 (0.875, 1.391) | 1.165 (0.945, 1.435) | 1.279 (1.039, 1.575) | 1.444 (1.176, 1.773) |  |
| 1 | 327 | 1.000 (Referent) | 0.984 (0.695, 1.393) | 0.985 (0.722, 1.343) | 1.041 (0.767, 1.412) | 1.185 (0.877, 1.600) |  |
| SOFA |  |  |  |  |  |  | <0.001 |
| 0.0-3.0 | 1336 | 1.000 (Referent) | 0.8617 (0.387, 1.921) | 1.251 (0.657, 2.384) | 1.080 (0.558, 2.090) | 1.180 (0.6071, 2.295) |  |
| 4.0-5.0 | 814 | 1.000 (Referent) | 1.140 (0.702, 1.852) | 1.315 (0.849, 2.037) | 1.308 (0.838, 2.042) | 1.069 (0.662, 1.726) |  |
| 6.0-9.0 | 1052 | 1.000 (Referent) | 0.969 (0.700, 1.342) | 1.143 (0.869, 1.503) | 1.214 (0.920, 1.601) | 1.217 (0.923, 1.605) |  |
| 10.0-23.0 | 791 | 1.000 (Referent) | 1.175 (0.908, 1.521) | 1.094 (0.862, 1.390) | 1.201 (0.952, 1.515) | 1.378 (1.098, 1.731) |  |
| SAPSII |  |  |  |  |  |  | <0.001 |
| 6.0-22.0 | 1040 | 1.000 (Referent) | 180.30 (0, Inf.) | 299.7 (0, Inf.) | 404.0 (0, Inf.) | 463.9 (0, Inf.) |  |
| 23.0-31.0 | 1074 | 1.000 (Referent) | 0.991 (0.684, 1.438) | 1.071 (0.779, 1.473) | 1.193 (0.865, 1.646) | 0.980 (0.688, 1.395) |  |
| 32.0-41.0 | 913 | 1.000 (Referent) | 1.030 (0.723, 1.468) | 1.245 (0.915, 1.694) | 1.243 (0.915, 1.689) | 1.205 (0.883, 1.644) |  |
| 42.0-98.0 | 966 | 1.000 (Referent) | 1.137 (0.840, 1.540) | 1.060 (0.798, 1.408) | 1.221 (0.926, 1.609) | 1.505 (1.147, 1.973) |  |
| LODS |  |  |  |  |  |  | <0.001 |
| 0.0-2.0 | 1212 | 1.000 (Referent) | 0.848 (0.387, 1.861) | 1.230 (0.658, 2.299) | 1.079 (0.561, 2.076) | 1.169 (0.611, 2.237) |  |
| 3.0-4.0 | 909 | 1.000 (Referent) | 0.972 (0.584, 1.619) | 1.155 (0.751, 1.777) | 1.228 (0.800, 1.885) | 1.079 (0.684, 1.703) |  |
| 5.0-7.0 | 998 | 1.000 (Referent) | 1.081 (0.752, 1.555) | 1.169 (0.850, 1.608) | 1.233 (0.895, 1.698) | 1.359 (0.992, 1.862) |  |
| 8.0-20.0 | 874 | 1.000 (Referent) | 1.144 (0.870, 1.504) | 1.111 (0.861, 1.432) | 1.229 (0.958, 1.577) | 1.398 (1.094, 1.788) |  |
| Sepsis |  |  |  |  |  |  | <0.001 |
| 0 | 1716 | 1.000 (Referent) | 1.632 (0.858, 3.104) | 1.548 (0.827, 2.896) | 1.645 (0.881, 3.071) | 1.757 (0.931, 3.314) |  |
| 1 | 2277 | 1.000 (Referent) | 1.008 (0.820, 1.239) | 1.133 (0.946, 1.359) | 1.280 (1.070, 1.530) | 1.511 (1.268, 1.801) |  |
| Myocardial infarct |  |  |  |  |  |  | 0.150 |
| 0 | 3634 | 1.000 (Referent) | 1.064 (0.876, 1.291) | 1.124 (0.944, 1.338) | 1.245 (1.048, 1.480) | 1.511 (1.276, 1.791) |  |
| 1 | 359 | 1.000 (Referent) | 631.3 (0, Inf.) | 396.8 (0, Inf.) | 411.8 (0, Inf.) | 482.3 (0, Inf.) |  |
| Congestive heart failure |  |  |  |  |  |  | 0.034 |
| 0 | 3382 | 1.000 (Referent) | 1.105 (0.888, 1.373) | 1.209 (0.994, 1.471) | 1.320 (1.086, 1.605) | 1.640 (1.353, 1.987) |  |
| 1 | 611 | 1.000 (Referent) | 1.012 (0.670, 1.528) | 0.969 (0.668, 1.407) | 1.206 (0.843, 1.726) | 1.219 (0.849, 1.749) |  |
| Peripheral vascular disease | |  |  |  |  |  | 0.282 |
| 0 | 3733 | 1.000 (Referent) | 1.109 (0.909, 1.352) | 1.159 (0.967, 1.389) | 1.294 (1.082, 1.548) | 1.548 (1.297, 1.847) |  |
| 1 | 260 | 1.000 (Referent) | 0.004 (0, Inf.) | 1.243 (0.684, 2.260) | 1.427 (0.786, 2.591) | 1.777 (0.984, 3.206) |  |
| Cerebrovascular disease |  |  |  |  |  |  | <0.001 |
| 0 | 3563 | 1.000 (Referent) | 1.019 (0.825, 1.258) | 1.130 (0.941, 1.357) | 1.258 (1.051, 1.507) | 1.526 (1.279, 1.820) |  |
| 1 | 430 | 1.000 (Referent) | 1.416 (0.779, 2.575) | 1.327 (0.735, 2.395) | 1.508 (0.832, 2.736) | 1.880 (1.034, 3.418) |  |
| Chronic pulmonary disease | |  |  |  |  |  | 0.056 |
| 0 | 3150 | 1.000 (Referent) | 1.161 (0.926, 1.457) | 1.196 (0.971, 1.473) | 1.353 (1.101, 1.662) | 1.609 (1.312, 1.972) |  |
| 1 | 843 | 1.000 (Referent) | 0.903 (0.618, 1.319) | 1.095 (0.800, 1.500) | 1.184 (0.864, 1.622) | 1.466 (1.080, 1.990) |  |
| Peptic ulcer disease |  |  |  |  |  |  | 0.471 |
| 0 | 3814 | 1.000 (Referent) | 1.080 (0.883, 1.320) | 1.167 (0.974, 1.397) | 1.316 (1.101, 1.572) | 1.586 (1.331, 1.891) |  |
| 1 | 179 | 1.000 (Referent) | 1.260 (0.641, 2.477) | 1.185 (0.622, 2.261) | 1.125 (0.571, 2.216) | 1.119 (0.572, 2.185) |  |
| Mild liver disease |  |  |  |  |  |  | <0.001 |
| 0 | 2373 | 1.000 (Referent) | 1.014 (0.703, 1.462) | 1.219 (0.890, 1.668) | 1.339 (0.980, 1.828) | 1.490 (1.090, 2.036) |  |
| 1 | 1620 | 1.000 (Referent) | 1.143(0.911, 1.434) | 1.199 (0.974, 1.477) | 1.339 (1.090, 1.645) | 1.558 (1.274, 1.906) |  |
| Severe liver disease |  |  |  |  |  |  | <0.001 |
| 0 | 3113 | 1.000 (Referent) | 1.132 (0.877, 1.462) | 1.160 (0.917, 1.467) | 1.262 (0.998, 1.596) | 1.455(1.151, 1.838) |  |
| 1 | 880 | 1.000 (Referent) | 1.063 (0.791, 1.429) | 1.274 (0.985, 1.647) | 1.477 (1.148, 1.900) | 1.708 (1.335, 2.185) |  |
| Renal disease |  |  |  |  |  |  | <0.001 |
| 0 | 3640 | 1.000 (Referent) | 1.076 (0.879, 1.317) | 1.155 (0.964, 1.384) | 1.292 (1.080, 1.547) | 1.556 (1.304, 1.856) |  |
| 1 | 353 | 1.000 (Referent) | 1.172 (0.605, 2.272) | 1.192 (0.637, 2.229) | 1.231 (0.668, 2.270) | 1.402 (0.758, 2.591) |  |
| Malignant cancer |  |  |  |  |  |  | <0.001 |
| 0 | 3698 | 1.000 (Referent) | 1.129 (0.903, 1.412) | 1.205 (0.983, 1.479) | 1.398 (1.143, 1.710) | 1.664 (1.363, 2.032) |  |
| 1 | 295 | 1.000 (Referent) | 1.021 (0.692, 1.507) | 1.081 (0.775, 1.508) | 0.988 (0.685, 1.426) | 1.282 (0.920, 1.786) |  |
| Metastatic solid tumor |  |  |  |  |  |  | <0.001 |
| 0 | 3889 | 1.000 (Referent) | 1.114 (0.915, 1.357) | 1.151 (0.9612, 1.379) | 1.319 (1.104, 1.575) | 1.573 (1.320, 1.874) |  |
| 1 | 104 | 1.000 (Referent) | 0.001 (0, Inf.) | 1.318 (0.698, 2.490) | 1.089 (0.534, 2.224) | 1.489 (0.771, 2.876) |  |

Abbreviations: AG, anion gap; CI, confidence interval; HR=hazard ratio; LODS, logistic organ dysfunction system; SAPSII, simplified acute physiology score II; SOFA, sequential organ failure assessment.

**Table S4. S**ubgroup analysis of the association between time-varying AG and 1-year mortality.

|  | N | AG<12 | 12≤AG<14 | 14≤AG<17 | 17≤AG<20 | AG≥20 | p for interaction |
| --- | --- | --- | --- | --- | --- | --- | --- |
|  |  | HR (95%CI) | HR (95%CI) | HR (95%CI) | HR (95%CI) | HR (95%CI) |  |
| Age |  |  |  |  |  |  | <0.001 |
| 18-55 | 2082 | 1.000 (Referent) | 1.064 (0.834, 1.358) | 1.114 (0.885, 1.401) | 1.237 (0.984, 1.555) | 1.463 (1.162, 1.842) |  |
| 56-94 | 1911 | 1.000 (Referent) | 1.087 (0.916, 1.289) | 1.122 (0.958, 1.313) | 1.236 (1.059, 1.443) | 1.359 (1.160, 1.591) |  |
| Sex |  |  |  |  |  |  | 0.017 |
| Female | 1073 | 1.000 (Referent) | 1.168 (0.878, 1.555) | 1.248 (0.954, 1.632) | 1.355 (1.035, 1.774) | 1.427 (1.088, 1.871) |  |
| Male | 2920 | 1.000 (Referent) | 1.042 (0.888, 1.224) | 1.050 (0.905, 1.219) | 1.173 (1.013, 1.358) | 1.363 (1.175, 1.580) |  |
| Ethnicity, n (%) |  |  |  |  |  |  |  |
| White | 2494 | 1.000 (Referent) | 1.059 (0.8796, 1.274) | 1.112 (0.939, 1.317) | 1.288 (1.089, 1.523) | 1.416 (1.193, 1.681) |  |
| Black | 359 | 1.000 (Referent) | 1.058 (0.644, 1.740) | 0.914 (0.583, 1.434) | 1.026 (0.666, 1.580) | 0.9852 (0.647, 1.500) |  |
| Hispanic | 167 | 1.000 (Referent) | 438.7 (0, Inf.) | 249.5 (0, Inf.) | 218.5 (0, Inf.) | 187.6 (0, Inf.) |  |
| Others | 973 | 1.000 (Referent) | 1.085 (0.856, 1.375) | 1.128 (0.901, 1.411) | 1.112 (0.888, 1.393) | 1.442 (1.154, 1.803) |  |
| Ventilation |  |  |  |  |  |  | <0.001 |
| 0 | 828 | 1.000 (Referent) | 0.744 (0.438, 1.263) | 1.017 (0.715, 1.449) | 1.101 (0.789, 1.536) | 0.8958 (0.619, 1.297) |  |
| 1 | 3165 | 1.000 (Referent) | 1.109 (0.956, 1.286) | 1.122 (0.977, 1.290) | 1.258 (1.095, 1.445) | 1.462 (1.272, 1.680) |  |
| Vasopression |  |  |  |  |  |  | <0.001 |
| 0 | 3666 | 1.000 (Referent) | 1.067 (0.910, 1.252) | 1.106 (0.955, 1.281) | 1.192 (1.030, 1.379) | 1.271 (1.096, 1.474) |  |
| 1 | 327 | 1.000 (Referent) | 0.998 (0.746, 1.335) | 0.921 (0.699, 1.212) | 1.012 (0.779, 1.316) | 1.103 (0.848, 1.435) |  |
| SOFA |  |  |  |  |  |  | <0.001 |
| 0.0-3.0 | 1336 | 1.000 (Referent) | 1.136 (0.808, 1.597) | 1.012 (0.702, 1.460) | 1.043 (0.733, 1.484) | 0.878 (0.582, 1.323) |  |
| 4.0-5.0 | 814 | 1.000 (Referent) | 1.201 (0.870, 1.658) | 1.179 (0.855, 1.627) | 1.261 (0.922, 1.725) | 0.950 (0.660, 1.366) |  |
| 6.0-9.0 | 1052 | 1.000 (Referent) | 0.911 (0.695, 1.195) | 1.155 (0.933, 1.429) | 1.199 (0.964, 1.490) | 1.174 (0.944, 1.460) |  |
| 10.0-23.0 | 791 | 1.000 (Referent) | 1.094 (0.879, 1.362) | 1.070 (0.879, 1.302) | 1.154 (0.954, 1.394) | 1.277 (1.057, 1.543) |  |
| SAPSII |  |  |  |  |  |  | <0.001 |
| 6.0-22.0 | 1040 | 1.000 (Referent) | 1.363 (1.175, 1.580) | 0.995 (0.597, 1.659) | 1.053 (0.648, 1.712) | 0.942 (0.577, 1.537) |  |
| 23.0-31.0 | 1074 | 1.000 (Referent) | 1.129 (0.875, 1.456) | 1.074 (0.839, 1.376) | 1.156 (0.894, 1.494) | 1.061 (0.821, 1.371) |  |
| 32.0-41.0 | 913 | 1.000 (Referent) | 1.104 (0.822, 1.482) | 1.296 (0.997, 1.684) | 1.285 (0.991, 1.667) | 1.199 (0.917, 1.568) |  |
| 42.0-98.0 | 966 | 1.000 (Referent) | 1.005(0.804, 1.257) | 0.966 (0.789, 1.182) | 1.102(0.908, 1.337) | 1.270 (1.043, 1.545) |  |
| LODS |  |  |  |  |  |  | <0.001 |
| 0.0-2.0 | 1212 | 1.000 (Referent) | 1.047 (0.738, 1.488) | 1.077 (0.763, 1.521) | 0.987 (0.688, 1.416) | 0.930 (0.629, 1.375) |  |
| 3.0-4.0 | 909 | 1.000 (Referent) | 1.147 (0.813, 1.619) | 1.190 (0.850, 1.666) | 1.261 (0.905, 1.757) | 1.100 (0.767, 1.576) |  |
| 5.0-7.0 | 998 | 1.000 (Referent) | 1.038 (0.794, 1.356) | 1.123 (0.889, 1.418) | 1.181 (0.935, 1.491) | 1.262 (1.004, 1.588) |  |
| 8.0-20.0 | 874 | 1.000 (Referent) | 1.064 (0.853, 1.327) | 1.028 (0.839, 1.260) | 1.154 (0.949, 1.404) | 1.251 (1.026, 1.525) |  |
| Sepsis |  |  |  |  |  |  | <0.001 |
| 0 | 1716 | 1.000 (Referent) | 1.281 (0.910, 1.803) | 1.153 (0.819, 1.625) | 1.335 (0.966, 1.845) | 1.232 (0.867, 1.753) |  |
| 1 | 2277 | 1.000 (Referent) | 1.016 (0.870, 1.186) | 1.104 (0.960, 1.270) | 1.205 (1.048, 1.386) | 1.370 (1.192, 1.575) |  |
| Myocardial infarct |  |  |  |  |  |  | 0.024 |
| 0 | 3634 | 1.000 (Referent) | 1.066 (0.922, 1.232） | 1.085 (0.948, 1.241) | 1.180 (1.032, 1.348) | 1.363 (1.191, 1.559) |  |
| 1 | 359 | 1.000 (Referent) | 1.126 (0.673, 1.884) | 1.303 (0.804, 2.112) | 1.647 (1.023, 2.652) | 1.546 (0.938, 2.548) |  |
| Congestive heart failure |  |  |  |  |  |  | <0.001 |
| 0 | 3382 | 1.000 (Referent) | 1.065 (0.898, 1.263) | 1.160 (0.995, 1.352) | 1.240 (1.064, 1.446) | 1.467 (1.258, 1.711) |  |
| 1 | 611 | 1.000 (Referent) | 1.054 (0.824, 1.348) | 0.906 (0.712, 1.153) | 1.130 (0.896, 1.426) | 1.080 (0.852, 1.370) |  |
| Peripheral vascular disease | |  |  |  |  |  | 0.022 |
| 0 | 3733 | 1.000 (Referent) | 1.090 (0.941, 1.264) | 1.111 (0.969, 1.275) | 1.223 (1.067, 1.402) | 1.374 (1.196, 1.577) |  |
| 1 | 260 | 1.000 (Referent) | 0.860 (0.518, 1.428) | 1.027 (0.689, 1.530) | 1.224 (0.849, 1.764) | 1.505 (1.034, 2.190) |  |
| Cerebrovascular disease |  |  |  |  |  |  | <0.001 |
| 0 | 3563 | 1.000 (Referent) | 1.005 (0.866, 1.167) | 1.068 (0.934, 1.222) | 1.185 (1.039, 1.351) | 1.340 (1.173, 1.530) |  |
| 1 | 430 | 1.000 (Referent) | 1.569 (0.886, 2.777) | 1.405 (0.794, 2.484) | 1.558 (0.867, 2.800) | 1.880 (1.034, 3.418) |  |
| Chronic pulmonary disease | |  |  |  |  |  | 0.030 |
| 0 | 3150 | 1.000 (Referent) | 1.097 (0.920, 1.307) | 1.155 (0.987, 1.352) | 1.267 (1.083, 1.482) | 1.432 (1.222, 1.679) |  |
| 1 | 843 | 1.000 (Referent) | 1.021 (0.811, 1.285) | 0.978 (0.777, 1.231) | 1.122 (0.898, 1.401) | 1.267 (1.011, 1.589) |  |
| Peptic ulcer disease |  |  |  |  |  |  | 0.600 |
| 0 | 3814 | 1.000 (Referent) | 1.065 (0.923, 1.228) | 1.089 (0.954, 1.244) | 1.222 (1.072, 1.392) | 1.382 (1.210, 1.578) |  |
| 1 | 179 | 1.000 (Referent) | 1.260 (0.641, 2.477) | 1.365 (0.752, 2.478) | 1.125 (0.571, 2.216) | 1.252 (0.667, 2.348) |  |
| Mild liver disease |  |  |  |  |  |  | <0.001 |
| 0 | 2373 | 1.000 (Referent) | 1.292 (0.957, 1.744) | 1.302 (0.963, 1.760) | 1.480 (1.104, 1.984) | 1.525 (1.123, 2.070) |  |
| 1 | 1620 | 1.000 (Referent) | 1.012 (0.860, 1.192) | 1.101 (0.953, 1.272) | 1.176 (1.018, 1.359) | 1.316 (1.141, 1.519) |  |
| Severe liver disease |  |  |  |  |  |  | <0.001 |
| 0 | 3113 | 1.000 (Referent) | 1.232 (0.999, 1.520) | 1.166 (0.947, 1.435) | 1.331 (1.089, 1.626) | 1.405 (1.140, 1.732) |  |
| 1 | 880 | 1.000 (Referent) | 0.931 (0.758, 1.142) | 1.155 (0.979, 1.364) | 1.221 (1.030, 1.447) | 1.399 (1.186, 1.649) |  |
| Renal disease |  |  |  |  |  |  | <0.001 |
| 0 | 3640 | 1.000 (Referent) | 1.106 (0.948 1.290) | 1.135 (0.983, 1.311) | 1.244 (1.077, 1.436) | 1.423 (1.231, 1.646) |  |
| 1 | 353 | 1.000 (Referent) | 0.859 (0.608, 1.214) | 0.879 (0.651, 1.186) | 0.956 (0.725, 1.261) | 0.976 (0.736, 1.295) |  |
| Malignant cancer |  |  |  |  |  |  | <0.001 |
| 0 | 3698 | 1.000 (Referent) | 1.114 (0.951, 1.306) | 1.143 (0.984, 1.328) | 1.282 (1.104, 1.488) | 1.459 (1.254, 1.698) |  |
| 1 | 295 | 1.000 (Referent) | 0.942 (0.685, 1.295) | 0.982 (0.749, 1.287) | 1.048 (0.800, 1.372) | 1.113 (0.847, 1.462) |  |
| Metastatic solid tumor |  |  |  |  |  |  | <0.001 |
| 0 | 3889 | 1.000 (Referent) | 1.083 (0.940, 1.248) | 1.085 (0.950, 1.238) | 1.217 (1.067, 1.387) | 1.376 (1.205, 1.570) |  |
| 1 | 104 | 1.000 (Referent) | 0.0008 (0, Inf.) | 1.407 (0.756, 2.617) | 1.368 (0.740, 2.531) | 1.489 (0.771, 2.876) |  |

Abbreviations: AG, anion gap; CI, confidence interval; LODS, logistic organ dysfunction system; SAPSII, simplified acute physiology score II; SOFA, sequential organ failure assessment.
